# Metabolomic and transcriptomic insights into the mechanism of sugar and acid accumulation in the pulp of sour- and sweet-tasting *Baccaurea ramiflora* Lour.

**DOI:** 10.3389/fpls.2025.1699388

**Published:** 2025-12-03

**Authors:** Jianjian Huang, Jie Chen, Yingchun Zhu, Xueying Wen, Kangyi Deng, Xiuxuan Lin, Hui Zhu, Yuzhong Zheng, Qinghan Wu, Yongqin Zheng, Fengnian Wu, Jean Wan Hong Yong

**Affiliations:** 1 School of Life Sciences and Food Engineering, Hanshan Normal University, Chaozhou, Guangdong, China; 2 College of Coastal Agricultural Sciences, Guangdong Ocean University, Zhanjiang, Guangdong, China; 3 Department of Biosystems and Technology, Swedish University of Agricultural Sciences, Alnarp, Sweden; 4 Brightlands Future of Farming Institute, Faculty of Science and Engineering, Maastricht University, Venlo, Netherlands

**Keywords:** *Baccaurea ramiflora* Lour., metabolomics, transcriptomics, sugarand acid accumulation, pulp

## Abstract

**Introduction:**

The taste differences between sour-tasting (LR) and sweet-tasting (BR) fruits of *Baccaurea ramiflora* Lour. are pronounced, but the underlying molecular mechanisms remain unclear.

**Methods:**

This study employed a combined metabolomic and transcriptomic analysis to elucidate the metabolic pathways governing this flavour variation.

**Results:**

Metabolomic profiling identified D-(+)-glucose and citric acid as the key taste determinants, with BR fruits exhibiting a significantly higher sugar-to-acid ratio than LR fruits. Transcriptomic data revealed that invertase (*INV*) activity correlated with D-glucose levels, whereas sucrose synthase (*SUS*) and sucrose-phosphate synthase (*SPS*) were associated with sucrose accumulation. In fully mature BR fruits, the suppressed expression of *INV* suggested that reduced sucrose hydrolysis contributes to their enhanced sweetness. Conversely, in LR fruits, elevated expression of hexokinase (*HK*) indicated higher glucose utilization. Furthermore, the expression of genes involved in organic acid metabolism—including citrate synthase (*CS*), aconitase (*ACO*), and NADP-malic enzyme (*NADP-ME*)—was found to regulate the content of citric and malic acids.

**Discussion:**

These findings advance our understanding of the molecular basis of flavour formation in *B. ramiflora*, and provide potential targets for the breeding and biotechnological improvement of fruit taste.

## 1 Introduction

*Baccaurea ramiflora* Lour., or Burmese grape, is an evergreen tree of the Phyllanthaceae family (order Malpighiales, APG IV system). It is native to South China, southern Yunnan Province, Southeast Asia, and South Asia (Li and Michael, 2008; Goyal et al., 2013). Its grape-like fruits have sweet-sour flesh, ideal for making juice, candied fruit, jam, and wine (Goyal et al., 2020; Rohilla, 2023). Unlike common fruit species, *B. ramiflora* shows distinctive sugar–acid composition, making it a suitable model for flavor metabolism. Its distinct soluble sugar-organic acid balance, unlike apple or citrus, enables exploration of metabolic bases of flavor variation. Soluble sugars and organic acids are key to fruit flavor, affecting their sensory quality (Borsani et al., 2009). Soluble sugars, including fructose, glucose, and sucrose, along with organic acids such as malic acid, citric acid, and oxalic acid, determine the sensory characteristics of the fruit (Pangborn, 1963). Organic acids typically accumulate during the early stages of fruit development and serve as respiratory substrates during fruit ripening (Diakou et al., 2000). Organic acid profiles vary across species. For instance, citrus fruits, strawberries (*Fragaria×ananassa*), mangoes (*Mangifera indica*), and cranberries (*Vaccinium macrocarpon* cv. Pilgrim) are rich in citric acid (Gil et al., 2000; Sadka et al., 2000; Celik et al., 2008), whereas apples, loquats, peaches, and grapes contain high concentrations of malic acid (Or et al., 2000; Chen et al., 2009).

Invertase (*INV*) hydrolyzes sucrose to glucose and fructose, whereas sucrose synthase (*SUS*) synthesizes sucrose from UDP-glucose and fructose (Miron and Schaffer, 1991; Granot et al., 2013). Hexokinase (*HK*) and fructokinase (*FK*) phosphorylate fructose and glucose to form fructose-6-phosphate (*F6P*) and glucose-6-phosphate (*G6P*), with phosphoglucose isomerase catalyzing the conversion between them (Umer et al., 2020). Acid invertases are central to plant development and growth (Ruan et al., 2010; Lu et al., 2017). Additionally, sucrose synthase (*SUS*) is involved in the accumulation of hexoses, with both sucrose and hexoses increasing during fruit development (Deluc et al., 2007).

Sugar and organic-acid metabolism converge via glycolysis to pyruvate (Wu et al., 2022). The distribution of organic acids in fruits is generally determined by the balance between synthesis, degradation, utilization, and compartmentation of acids (Diakou et al., 2000). Organic acids are prevalent intermediates in the tricarboxylic acid (TCA) cycle, playing a role in aerobic cellular respiration. Enzymes involved in this biochemical pathway include phosphoenolpyruvate carboxylase (PEPC, EC 4.1.1.31), citrate synthase (CS, EC 4.1.3.7), aconitase (ACO, EC 4.2.1.3), malate dehydrogenase (MDH, EC 1.1.1.37), and malic enzyme (ME, EC 1.1.1.40) (Sadka et al., 2000; Wu et al., 2022). Malate synthesis primarily occurs in the cytoplasm, catalyzed by PEPC and NAD-dependent malate dehydrogenase (Moing et al., 2000). The concentration of malate rapidly decreases during fruit ripening, generally attributed to degradation by cytoplasmic NADP-dependent malic enzyme (NADP-ME, EC 1.1.1.40) (Hirai and Ueno, 1997). Conversely, citrate biosynthesis and breakdown, besides cytoplasmic ACO, are mediated through mitochondrial CS, NAD-dependent isocitrate dehydrogenase (NAD-IDH, EC 1.1.1.41), and aconitate hydratase (ACO, EC 4.2.1.3) (Kubo et al., 2002). In the synthesis of tartaric acid, the substrate l-ascorbic acid is converted into l-tartaric acid through l-idonate dehydrogenase (l-IdnDH, EC 1.1.1.264) and other unknown enzymes (Saito and Kasai, 1969).

Extensive research has been conducted on the metabolism of soluble sugars and organic acids in plants using multi-omics approaches, with significant findings reported for crops such as Ponkan fruit, watermelon fruit, apple, grape, and sweet orange (Shangguan et al., 2015; Yao et al., 2018; Umer et al., 2020). *B. ramiflora’s* distinctive traits make it a useful model for studying fruit metabolism. Unlike common fruit species, it exhibits an unusual sugar composition, with a high ratio of organic acids, such as malic and citric acid, which may be linked to its ecological adaptation and fruit characteristics. Additionally, the fruit’s remarkable potential for processing into various products, such as juice and wine, emphasizes its importance in both ecological and market contexts. These distinctive features provide a valuable opportunity to explore how its unique metabolic processes influence flavor formation, offering insights into the broader implications of sugar-acid metabolism in fruit development. Despite these traits, studies on *B. ramiflora* remain limited, mainly focusing on compositional analysis and basic metabolomics (Pandey et al., 2018; Chen et al., 2023). The molecular mechanisms behind the flavor formation of *B. ramiflora* and its metabolic processes, especially in relation to sugar and acid metabolism, remain largely unexplored. We address this gap by examining how sugar and organic-acid metabolism shapes *B. ramiflora* flavor. What specific molecular mechanisms are involved in this process? By conducting parallel metabolomics and transcriptomics, we aim to deeply explore these metabolic pathways and their impact on flavor formation, revealing the associated molecular mechanisms. Understanding sugar–acid metabolism in *B. ramiflora* has implications for breeding, food science, and processing. Insights from this study can inform breeding programs aimed at enhancing fruit flavor, nutritional content, and shelf life, ultimately contributing to better consumer products and health benefits.

## 2 Materials and methods

### 2.1 Plant materials

**Figure 1:**
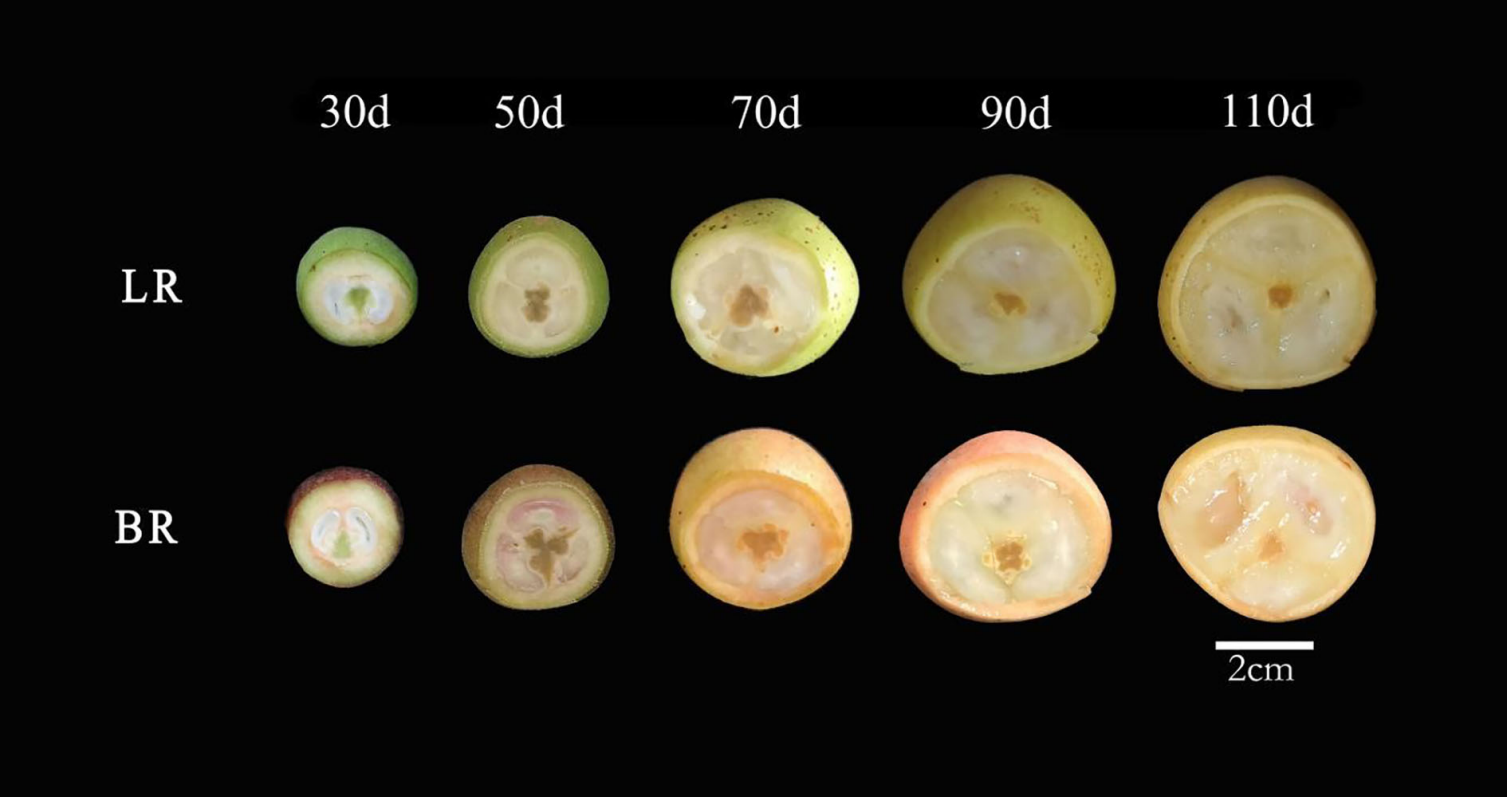
Five fruit profiles of the maturation process of *Baccaurea ramiflora* in LR and BR. From left to right, 30, 50, 70, 90 and 110d after the full flowering (DAF), respectively. Scale bar = 2 cm.

Two local varieties of *B. ramiflora* were studied: BR (off-white skin, pink flesh, sweeter) and LR (pale-green skin, off-white flesh, more acidic) (**Figure 1**). The BR variety is noted for its sweeter taste compared to LR. Both local varieties are germplasm resources that have been selected and preserved by local communities over many years and are now widely cultivated from Fangchenggang to Chongzuo in Guangxi, China. The experimental materials were collected from *B. ramiflora* trees cultivated by villagers in Nasuo Town, Fangcheng District, Fangchenggang (N 21°42′33″, E 108°6′29″, alt 20 m). The sampled trees were artificially cultivated, with an age of over 10 years, in good health, and at their peak fruit-bearing stage. To ensure the representativeness of the samples and the comparability of experimental data, the selected trees met the following criteria: (1) similar age (15–20 years), (2) vigorous growth with no visible diseases or physiological defects, (3) consistent environmental conditions, including soil type, light exposure, and cultivation management, and (4) well-balanced tree structure with abundant foliage and stable fruit-bearing capacity.

Samples from LR and BR varieties were collected at five different stages of fruit maturation and development (30, 50, 70, 90, and 110 days after full bloom [DAF]) (**Figure 1**). For each local variety, 6 mature fruits of consistent quality were collected from three independent, well-grown and balanced trees (i.e., biological replicates), totaling 18 fruits of the same variety mixed together. Then, 4 fruits of the same variety were randomly selected as one biological sample, with 3 biological replicates in total. Each biological replicate was derived from independent trees to minimize potential tree-to-tree variation and ensure that biological differences were properly represented.

To further ensure the reliability and consistency of the data, environmental conditions, including soil type, light exposure, and microclimate factors (e.g., temperature, humidity), were recorded during the collection period. Samples were immediately frozen in liquid nitrogen upon field collection and stored at -80 °C in the laboratory for further use and were subsequently utilized for metabolomic and transcriptomic sequencing analyses. The voucher specimen of the fruits was deposited in the Plant Research Laboratory of Hanshan Normal University, Chaozhou, Guangdong, China (No: BR01-BR60, LR01-LR60).

### 2.2 Identification of soluble sugars and organic acids by UPLC-MS/MS

Non-targeted metabolomic analysis of *B. ramiflora* pulp was performed using UPLC-MS/MS technology. Metabolite identification was based on a standard pipeline: first, preliminary identification was achieved by comparing the retention times of metabolites in the samples with our laboratory’s self-built standard database; second, UPLC-MS/MS fragmentation patterns were matched against public mass spectral databases such as HMDB and MassBank, and metabolites with a similarity score greater than 70% were adopted; for metabolites lacking reference standards or database information, putative identification was conducted by combining their accurate molecular weight (error <no><</no> 5 ppm) with predicted fragmentation patterns. All identifications were manually verified for chemical plausibility and biological relevance. Metabolites were statistically analyzed using the metabolomics R software package metaX developed by BGI-Research, which includes metabolite classification annotation and functional annotation (Wen et al., 2017). Principal Component Analysis (PCA) was employed to reduce the dimensionality of multivariate raw data, analyzing similarities and differences within and between sample groups, as well as identifying outliers (to detect any abnormal samples). The Partial Least Squares-Discriminant Analysis (PLS-DA) model was used to calculate the importance of variable projection (VIP) for the 2 principal components. VIP scores assess the impact strength and explanatory power of each metabolite’s expression pattern on the classification discrimination of sample groups, aiding in the selection of metabolic biomarkers. Data were first log-transformed using base 2, followed by the establishment of the PLS-DA model with scaling method ‘Par’; the model underwent 7 rounds of cross-validation and 200 rounds of response permutation testing (RPT) to evaluate model quality. The resulting model parameters (*R*² = 0.89 and *Q*² = 0.82) indicated excellent explanatory and predictive capability, respectively. Furthermore, the permutation test *p* -value was 0.02 (*p* < 0.05), confirming that the model was robust and not overfitted. Differential metabolites were selected based on the fold change (FC) obtained from univariate analysis and the results of the student’s t-test. Both PCA and FC were processed with log2 transformation, with selection criteria set at *p*-value < 0.05, VIP ≥ 1, and FC ≥ 1.2 or ≤ 0.83, and FDR < 0.05, with the conclusions remaining robust after correction. UPLC-MS/MS-based metabolomics enables comprehensive metabolite detection in fruit while ensuring result accuracy through rigorous statistical analysis.

### 2.3 RNA library construction and sequencing

Total RNA was extracted according to the method reported in (Zhang et al., 2020). The resulting RNA samples exhibited concentrations greater than 500 ng/µL, a 28S/18S ratio exceeding 1.8, and RNA integrity number (RIN) values ranging from 8.5 to 9.0, indicating high-quality RNA suitable for downstream analyses. Agarose gel electrophoresis: The integrity of RNA was assessed by observing clear bands of 28S and 18S rRNA. Clear bands with the intensity of the 28S band being twice that of the 18S band indicate good RNA integrity. The purity of RNA samples is measured using UV spectrophotometry, ensuring the absence of protein, polysaccharides, or other contaminants. The total RNA concentration is typically determined using a colorimetric method (NanoDrop).

The RNA-seq library sequencing process included: total RNA sample extraction, sample testing, RNA library construction, library quality control, and sequencing. Fruit pulp mRNA was enriched using magnetic beads attached to Oligo(dT); fragmentation buffer was then added to break the mRNA into 200 nt short fragments. Using mRNA as a template, the first strand of cDNA was synthesized with 6-base random primers for reverse transcription, followed by the addition of buffer, dNTPs, RNase H, and DNA polymerase I to synthesize the second cDNA strand. The double-stranded cDNA underwent end repair and adenylation at the 3’ end. The cDNA was purified using the QiaQuick PCR purification kit and eluted with EB buffer, followed by end repair and adapter ligation. The library was then size-selected using agarose gel electrophoresis and enriched with PCR to amplify the cDNA. In total, 30 *B. ramiflora* pulp libraries were constructed, 15 each for BR and LR, with three biological replicates for each developmental stage at 30, 50, 70, 90, and 110 DAF. After library construction, initial nucleic acid quantification was performed using the Qubit 2.0 fluorometer, followed by Agilent 2100 analysis to assess the insert size of the libraries. Once the insert size met expectations, the effective concentration of the libraries was accurately quantified using real-time fluorescence quantitative PCR to ensure the quality of the *B. ramiflora* pulp transcriptome sequencing libraries. After passing quality control, the libraries were sequenced on the Illumina Novaseq platform with PE150 sequencing, with library construction and sequencing services provided by BGI-Research.

The cDNA libraries were sequenced on the Illumina NovaSeq platform to generate paired-end raw reads. These raw data were first subjected to quality control using FASTQC, which involved removing adapter sequences and low-quality reads to obtain high-quality clean data. On average, each library yielded approximately 6.5 Gb of clean data, ensuring sufficient depth for subsequent transcriptomic analysis. The clean reads were then aligned to the *B. ramiflora* reference genome from Huang et al., 2024 (Huang et al., 2024), achieving an average mapping rate of 88.5%, which indicates a high level of compatibility between the sequencing data and the genome. The reference genome itself is of high quality, with a scaffold N50 of 1.2 Mb and a BUSCO completeness score of 96.5% (embryophyta_odb10), confirming its suitability for RNA-seq read mapping and gene annotation. This integrated bioinformatics analysis focused on gene expression quantification, identification of differentially expressed genes (DEGs), transcription factor analysis, and functional enrichment analysis of DEGs in Gene Ontology (GO) and Kyoto Encyclopedia of Genes and Genomes (KEGG) pathways across different fruit pulp developmental stages.The raw data obtained from sequencing contained adapter sequences, duplicates, and low-quality reads, which could affect subsequent analysis and alignment. The raw data were filtered with the following criteria: paired reads were removed if one read contained more than 3 Ns; paired reads were removed if one read had a quality score below 5 for more than 20% of its bases; adapter sequences required at least an 8bp match for removal. This process yielded clean, high-quality reads for further data analysis. Quality assessment was based on the proportion of Q_20_ and Q_30_ scores, with higher values (typically ≥85%) indicating higher quality of the post-sequencing clean reads.

### 2.4 Differential gene expression analysis

Differential expression of genes across samples was analyzed using the EdgeR software (Smyth, 2010), calculating the *p*-value and adjusted *p*-value (*p-adj*) for differential expression. The *p-adj*, a corrected *p*-value, signifies the significance of gene expression differences, with smaller values indicating more significant differences. The selection criteria were set as: *p-adj* < 0.05 & |log_2_FoldChange| > 1. If the number of DEGs was low, the parameters were adjusted to *p*-value < 0.05 & |log_2_FoldChange| > 1 for selection. The input data for the differential gene expression analysis were the read count data obtained from gene quantification analysis, involving three main steps: normalization of read count data; calculation of hypothesis testing probability (*p*-value) based on the model; and correction for multiple hypothesis testing to obtain the *p-adj* value (false discovery rate).

Enrichment analysis was performed on the corrected DEGs using the topGO software for GO enrichment analysis of DEGs (Hong et al., 2014) and the Kobas software for KEGG pathway enrichment analysis of DEGs. A *p*-value < 0.05 was considered significant for GO and KEGG pathway enrichment, used to analyze the relationship between differentially expressed genes and related biological functions. Enrichment pathways provided further insight into the signaling pathways and specific biological functions involved in gene metabolism.

### 2.5 Transcription factor analysis

Transcription factors play a pivotal role in regulating the binding of RNA polymerase to the DNA template, thereby controlling the transcription process. A comprehensive understanding of the gene transcription process can be achieved through the analysis of transcription factors. For the prediction of transcription factors, the iTAK software is employed, which utilizes Hmmscan to annotate and identify transcription factors (TFs) based on their Pfam families’ files (Paulino et al., 2010).

### 2.6 qRT-PCR validation

To verify the accuracy of RNA-seq data for different samples of the *B. ramiflora* fruit flesh, this study selected 19 DEGs for analysis via quantitative real-time PCR (qRT-PCR). These DEGs include genes involved in the flavonoid biosynthesis pathway, sugar metabolism pathway, and transcription factors. The first strand of cDNA was synthesized using the Servicebio^®^ RT First Strand cDNA Synthesis Kit (Servicebio G3330) with reverse transcriptase. Subsequently, PCR amplification was performed on the reverse-transcribed cDNA. The housekeeping gene gapdh was used as an internal reference. Primers for the differentially expressed genes were designed using Primer 6.0, and their sequences are provided in **Supplementary Table S1**.

### 2.7 Data analysis

Statistical analysis and graphical representation of the data were performed using SPSS software (v22.0, IBM Corp., Armonk, NY, USA) and OriginLab software (v2019, OriginLab Inc., Northampton, MA, USA), with results expressed as mean ± standard deviation (SD). The data were subjected to one-way analysis of variance (ANOVA) and further analyzed using Tukey’s honestly significant difference (HSD) test to determine statistical significance (*p* < 0.05).

## 3 Results

### 3.1 PCA analysis

**Figure 2:**
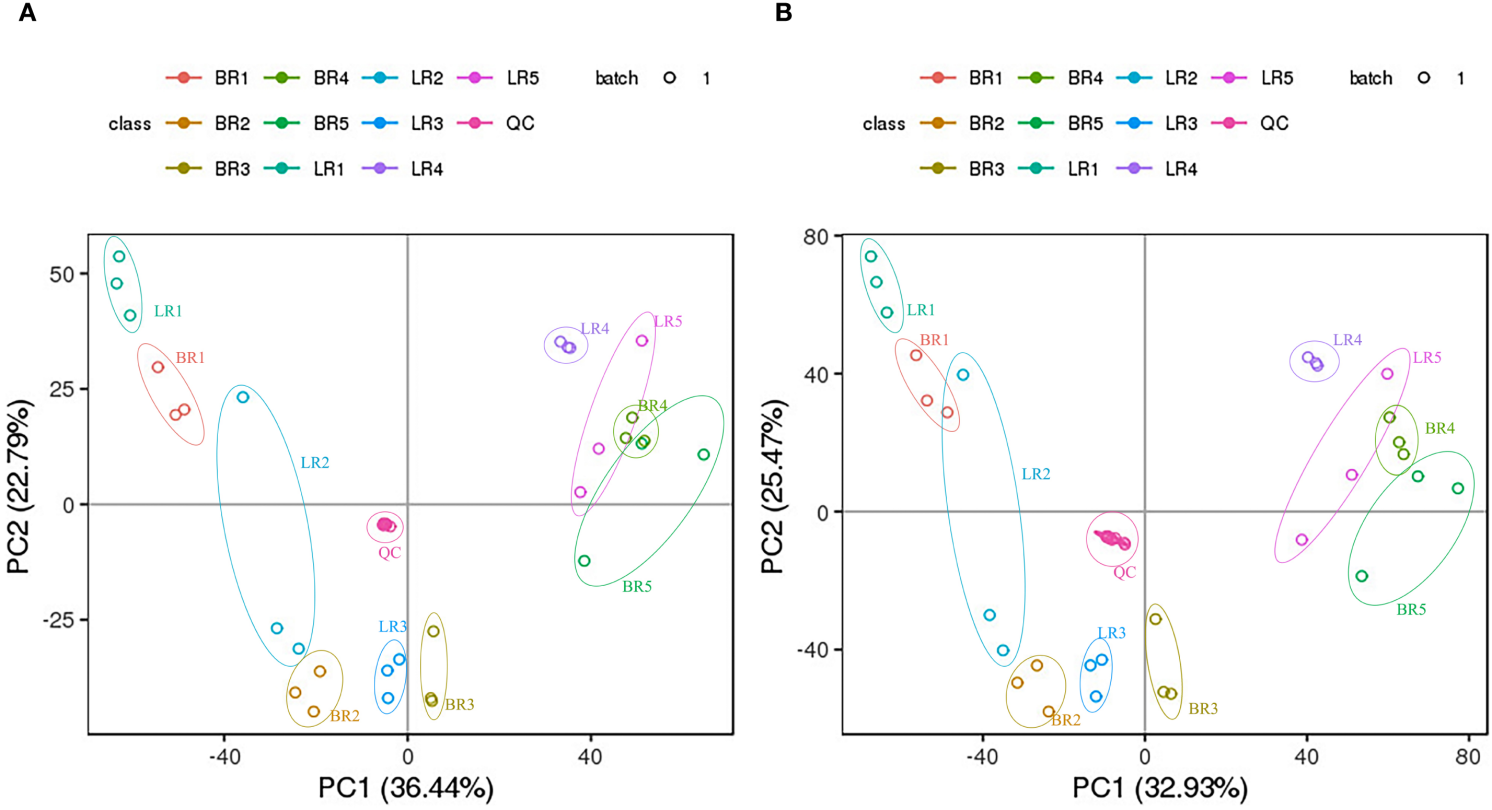
PCA analysis of metabolites identified from Baccaurea ramiflora ‘LR’ and ‘BR’. Equal volumes of ‘LR’ and ‘BR’ fruit samples were mixed for use as aquality control (QC). **(A)** Left panel shows the positive ion mode; **(B)** Right panel shows the negative ion mode.

Logarithmic transformation (base 2) was applied to the peak areas of metabolites detected in both positive and negative ionization modes. Subsequently, PCA was performed to evaluate metabolic patterns and sample clustering. PCA separated BR, LR, and QC (*p*<0.01); replicates clustered tightly. (**Figure 2**), and biological replicates of samples from each developmental stage clustered well together. This analysis uncovered 2 distinct groups associated with LR and BR, respectively. Thus, the PCA indicates that these 2 varieties possess different metabolic characteristics.

### 3.2 Dynamic changes in sugars and organic acids in *B. ramiflora* pulp

**Figure 3:**
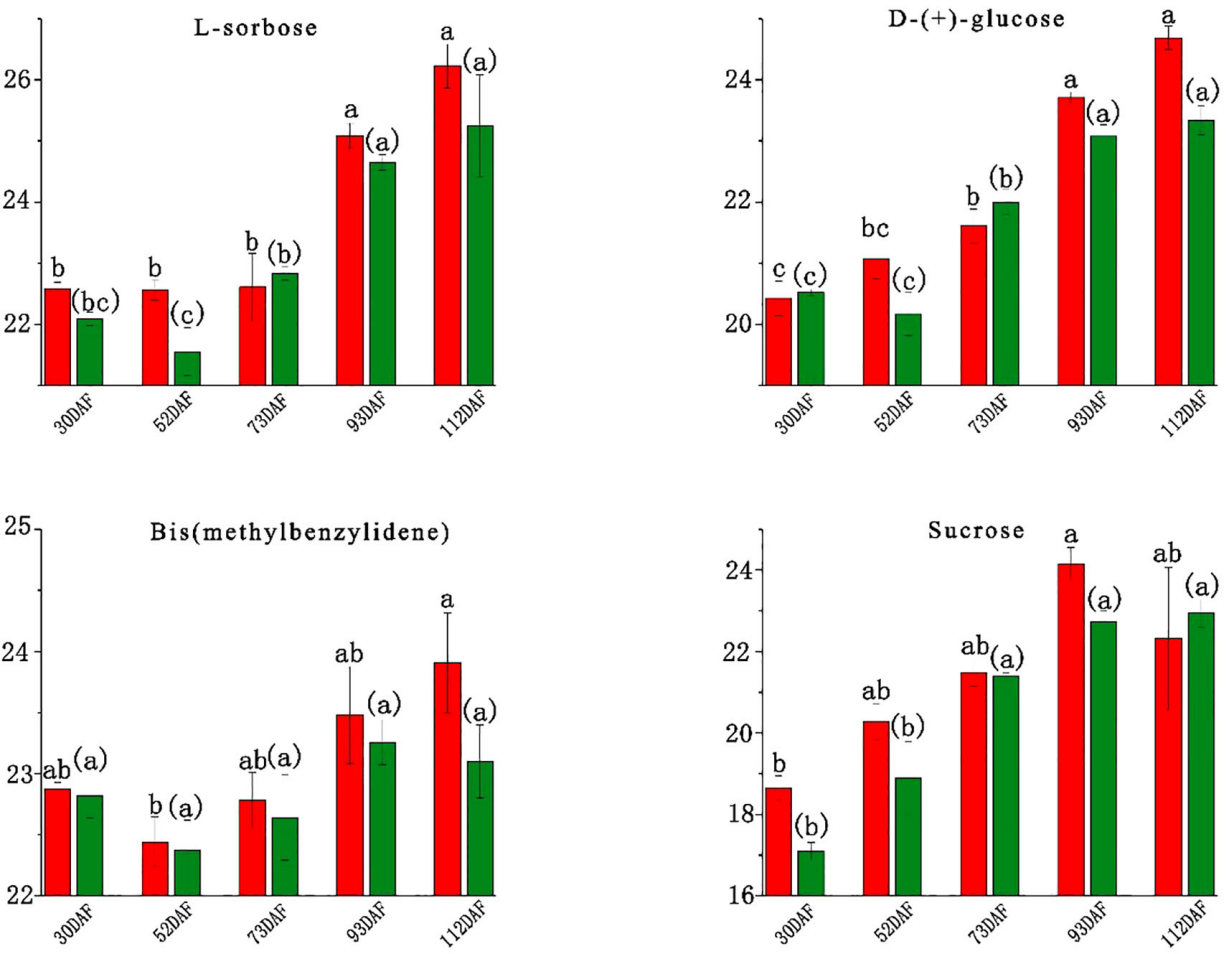
Soluble sugar content of different development stage in *Baccaurea ramiflora* fruit. The abscissa represents the number of days after the full flowering of *B. ramiflora*, and the ordinate represents the peak area of metabolites after Log2 treatment. Red represents BR and green represents LR (The same as below). Different lowercase letters (a, b, c) above the bars indicate statistically significant differences (P < 0.05) as determined by one-way ANOVA followed by Tukey’s HSD test. Bars sharing the same letter are not significantly different, whereas bars with different letters are significantly different.

Fruit taste primarily reflects sugar and organic-acid composition, primarily determined by soluble sugars and organic acids (Wang et al., 2009). In *B. ramiflora* fruit, 12 carbohydrates were identified, including Nystose, Gluconic acid, Δ-gluconic acid δ-lactone, L-Sorbose, D-(+)-glucose, Fructose, Lusitanicoside, Uridine 5’-diphosphogalactose, Sibiricose A3, 10-hydroxyligustroside, Bis(methylbenzylidene)sorbitol, and Zeatin-7-n-glucoside (**Supplementary Table S3**). Major sugars were L-sorbose, D-(+)-glucose, bis(methylbenzylidene)sorbitol, and sucrose; fructose was not detected. The content changes of these four carbohydrates across 5 different maturity stages are shown in **Figure 3**. L-sorbose increased during maturation, with a marked rise at 90 DAF, indicating the onset of ripening, reaching its highest content at full maturity at 110 DAF; only BR4 vs BR3 showed a significant increase (P 0.033, FC 2.327, VIP 1.301). D-(+)-glucose followed a similar pattern, peaking near maturity, sharply increasing at 90 DAF; significant increases were observed in LR3 vs LR2 (P 0.017, FC 1.780, VIP 1.170) and BR4 vs BR3 (P 0.011, FC 2.056, VIP 1.122), but a significant decrease in LR5 vs BR5 at full maturity (P 0.013, FC -1.334, VIP 1.129). The sharp increase in L-sorbose and D-(+)-glucose content at 90 DAF suggests that *B. ramiflora* fruits start to accumulate sugars significantly at 70 DAF, entering the ripening stage. Bis(methylbenzylidene)sorbitol remained stable, with no significant changes between adjacent developmental stages, indicating its minimal impact on pulp taste. Sucrose content was the lowest, generally increasing during development with a slight decrease at BR5, which was not significant, suggesting a faster conversion of sucrose to glucose in BR *B. ramiflora* from 90 DAF to 110 DAF compared to LR; only BR4 vs BR3 showed a significant increase (P 0.008, FC 2.697, VIP 1.148), further indicating the onset of maturation from 70 DAF. Importantly, at full maturity (110 DAF), only the concentration of the main soluble sugar (D-(+)-glucose) significantly decreased in LR, which may determine the superior taste of BR *B. ramiflora* pulp.

**Figure 4:**
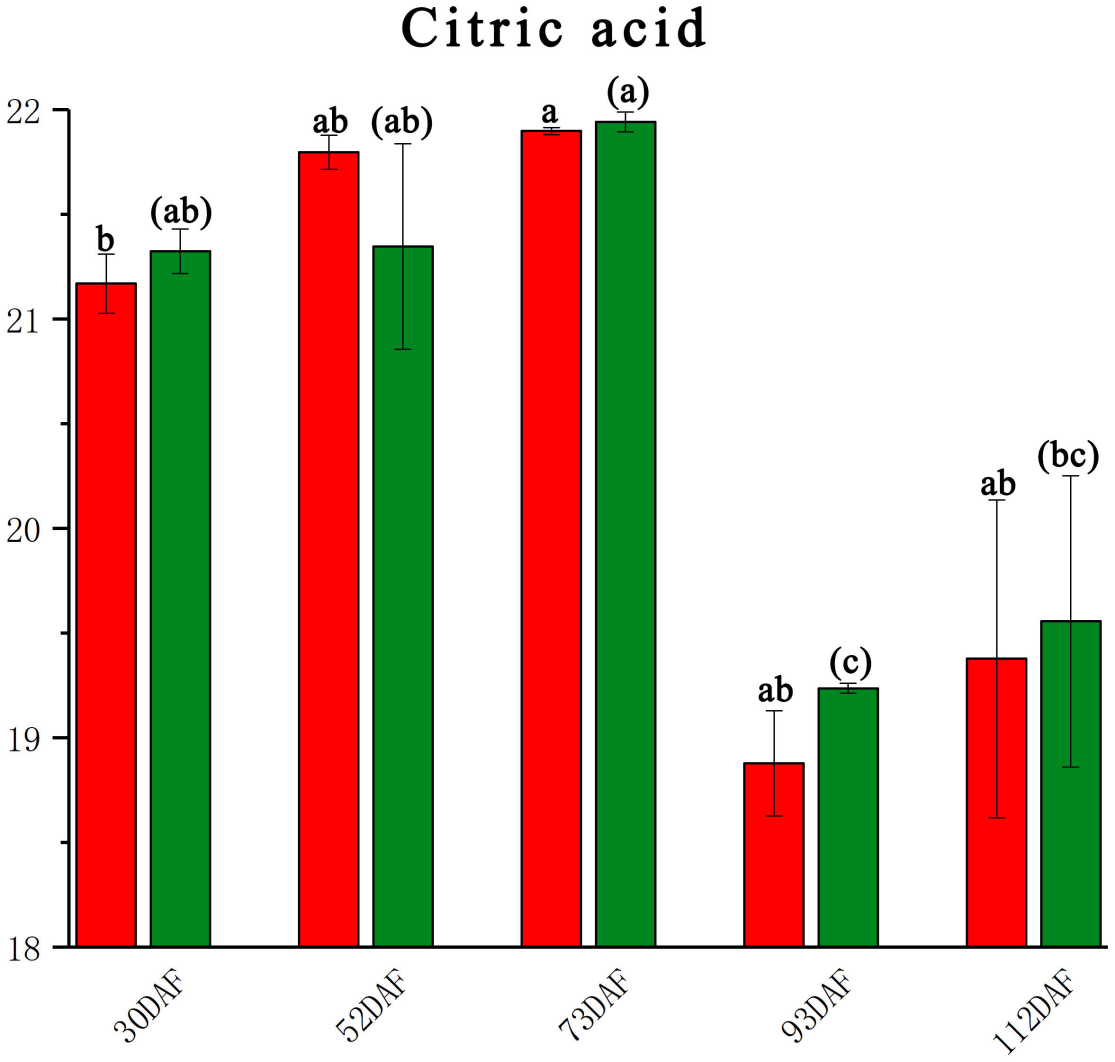
Citric acid content of different development stage in *Baccaurea ramiflora* fruit. Red represents BR and green represents LR. Different lowercase letters (a, b, c) above the bars indicate statistically significant differences (P < 0.05) as determined by one-way ANOVA followed by Tukey’s HSD test. Bars sharing the same letter are not significantly different, whereas bars with different letters are significantly different.

In the pulp of the *B. ramiflora*, citric acid was the predominant organic acid, while malic acid and oxalic acid were not detected. The trend of citric acid levels first increased and then decreased (**Figure 4**), aligning with the taste development of *B. ramiflora* pulp through its developmental stages—from astringent to sour and finally to sweet. Significant decreases were observed in the development process between LR4 vs LR3 (P 0.00002, FC -2.707, VIP 1.309) and BR4 vs BR3 (P 0.007, FC -2.974, VIP 1.708), indicating that *B. ramiflora* fruits began to enter the ripening stage at 70 DAF, consistent with the changes in soluble sugars and the maturation process. Citric acid did not differ significantly between BR and LR across stages, suggesting that citric acid does not influence the taste of *B. ramiflora* pulp at full maturity in either variety. At 110 DAF, BR had higher total sugars and a higher sugar-to-acid ratio, which may partly explain the sweeter taste of BR’s pulp.

### 3.3 Structural genes in the sugar and acid metabolic pathways of *B. ramiflora* pulp

**Figure 5:**
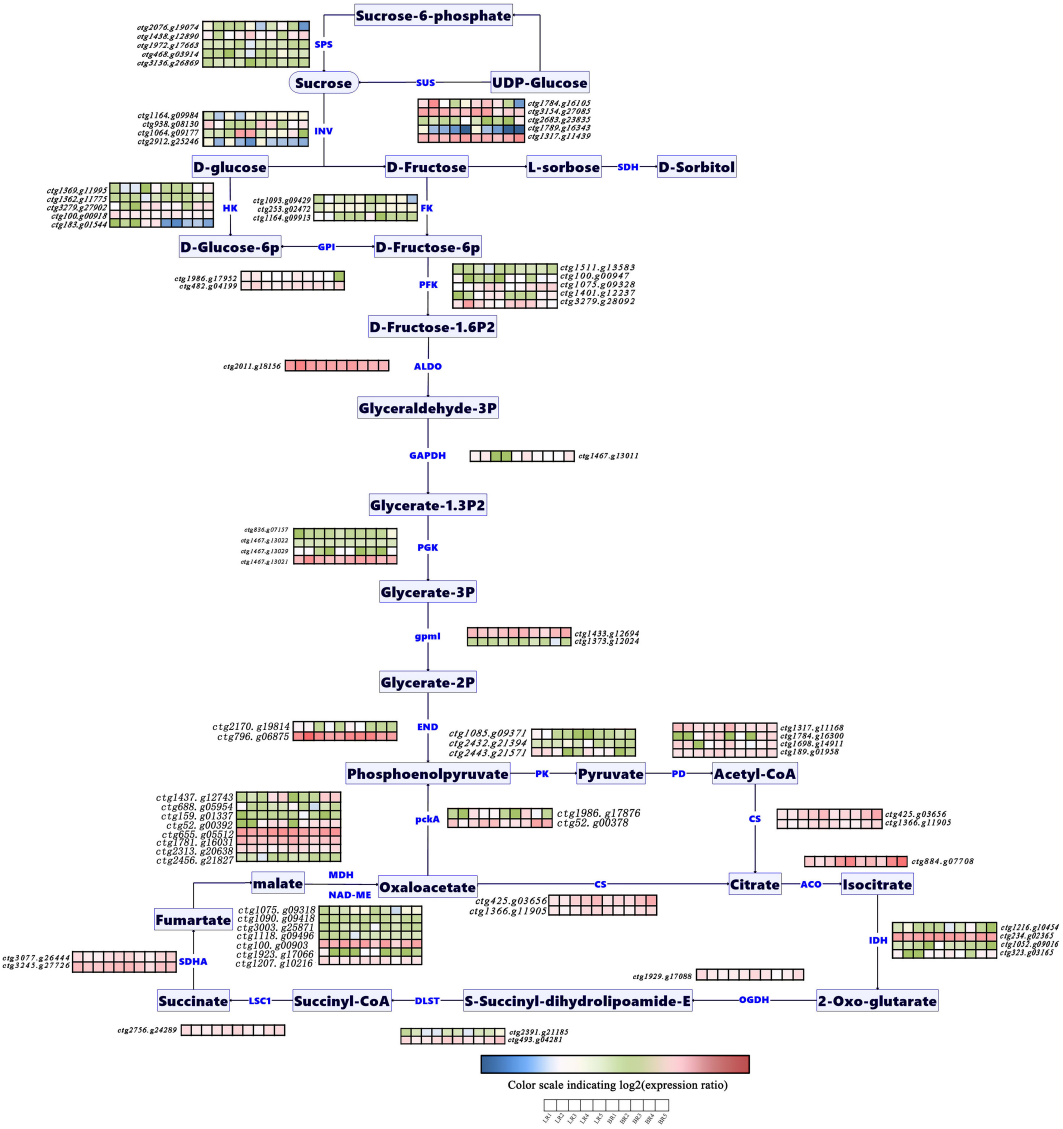
Expression level of genes related to soluble sugar and organic acid metabolism pathway during pulp development in *Baccaurea ramiflora.* The heatmap represents the expression levels of the listed structural genes in the two types of *B. ramiflora*. The color changes from blue to red indicate that the gene expression levels are from low to high, and the same applies below.

Ongoing studies on sugar and acid metabolic pathways across various plant fruits (Ruan et al., 2010; Shangguan et al., 2015) have led to our investigation into the DEGs during the development of *B. ramiflora* pulp. GO enrichment and KEGG pathway analyses facilitated the identification of structural genes within these pathways and their differential expression patterns across developmental stages (**Supplementary Table S4**). We integrated pathway schematics with a heatmap of structural-gene expression (**Figure 5**). Seventy-nine structural genes were identified; 37 were differentially expressed. (**Supplementary Table S4**).

For soluble sugar metabolism, structural genes include *INV* (Invertase) (ctg1164.g09984, ctg938.g08130, ctg1064.g09177, and ctg2912.g25246), *SUS* (Sucrose Synthase) (ctg1784.g16105, ctg3154.g27085, ctg2683.g23835, and ctg1317.g11439), *SPS* (Sucrose-Phosphate Synthase) (ctg2076.g19074, ctg1438.g12890, ctg1972.g17663, ctg468.g03914, and ctg3136.g26869), *HK* (Hexokinase) (ctg1369.g11995, ctg1362.g11775, ctg3279.g27902, ctg100.g00918, ctg183.g01544), *FK* (Fructokinase) (ctg1093.g09429, ctg253.g02472, and ctg1164.g09913), *GPI* (Glucose-6-Phosphate Isomerase) (ctg1986.g17952 and ctg482.g04199), *FBP* (Fructose-1,6-Bisphosphatase I) (ctg1968.g17584 and ctg2513.g22492), *PFK9* (6-Phosphofructokinase) (ctg1511.g13583, ctg100.g00947, ctg1075.g09328, ctg1401.g12237, and ctg3279.g28092), and *ALDO* (Fructose-Bisphosphate Aldolase) (ctg2011.g18156).

The pathway then transitions into glycolysis, with structural genes including *GAPDH* (Glyceraldehyde 3-Phosphate Dehydrogenase) (ctg1467.g13011), *PGK* (Phosphoglycerate Kinase) (ctg836.g07157, ctg1467.g13022, ctg1467.g13029, and ctg1467.g13021), *gpmI* (Phosphoglycerate Mutase) (ctg1433.g12694 and ctg1373.g12024), *ENO* (Enolase) (ctg2170.g19814 and ctg796.g06875), *pckA* (Phosphoenolpyruvate Carboxykinase (ATP)) (ctg1986.g17876 and ctg52.g00378), *PK* (Pyruvate Kinase) (ctg1085.g09371, ctg2432.g21394, and ctg2443.g21571), and *PD* (Pyruvate Dehydrogenase) (ctg1317.g11168, ctg1784.g16300, ctg1698.g14911, and ctg189.g01958).

Finally, entering the organic acid metabolism pathway, structural genes include *CS* (Citrate Synthase) (ctg425.g03656 and ctg1366.g11905), *ACO* (Aconitate Hydratase) (ctg884.g07708), *IDH* (Isocitrate Dehydrogenase) (ctg1216.g10454, ctg234.g02365, ctg1052.g09016, and ctg323.g03165), *OGDH*, *sucA* (2-Oxoglutarate Dehydrogenase E1 Component) (ctg2391.g21185 and ctg493.g04281), *LSC1* (Succinyl-CoA Synthetase Alpha Subunit) (ctg2756.g24289), *SDHA*, *SDH1* (Succinate Dehydrogenase (Ubiquinone) Flavoprotein Subunit) (ctg3077.g26444 and ctg3245.g27726), *fumA*, *fumB* (Fumarate Hydratase) (ctg1317.g11423), and *MDH1* (Malate Dehydrogenase) (ctg1437.g12743, ctg688.g05954, ctg159.g01337, ctg52.g00392, ctg655.g05512, ctg1781.g16031, ctg2313.g20638, and ctg2456.g21827), *NADP-ME* (NAD-Dependent Malic Enzyme) (ctg1075.g09318, ctg1090.g09418, ctg3003.g25871, ctg1118.g09496, ctg100.g00903, ctg1923.g17066, and ctg1207.g10216).

This comprehensive analysis of structural genes involved in the sugar and acid metabolic pathways provides a foundational understanding of the molecular mechanisms underlying the development and flavor profiles of *B. ramiflora* pulp.

### 3.4 Differential expression of genes in sugar and acid metabolic pathways

Differential expression analysis of genes involved in soluble sugar and organic acid metabolic pathways was conducted across five developmental stages of *Baccaurea ramiflora* fruits from both light (LR) and brown (BR) regions (**Supplementary Table S2**). The results revealed dynamic and stage-specific transcriptional regulation associated with fruit maturation and flavor development.

Stage I (young fruit stage): In the sugar metabolism pathway, *HK* (ctg183.g01544) and *PFK9* (ctg1401.g12237) were found upregulated, facilitating the conversion of glucose to D-fructose-1,6-bisphosphate (D-fructose-1,6-P_2_). Within the glycolytic pathway, *pckA* (ctg1986.g17876 and ctg52.g00378) showed increased expression, whereas *PK* (ctg2432.g21394) was downregulated. In the organic acid metabolism, *IDH* (ctg1216.g10454) was found downregulated, indicating a potential reduction in tricarboxylic acid cycle activity at this stage.

Stage II (expanding fruit stage): The sugar metabolism pathway exhibited a transcriptional shift characterized by suppressed expression of *INV* (ctg2912.g25246) and elevated expression of *SPS* (ctg2076.g19074) and *FK* (ctg1093.g09429).Among *HK* genes, ctg3279.g27902 was downregulated, while ctg183.g01544 was upregulated, collectively favoring enhanced sucrose biosynthesis and the rapid conversion of fructose into D-fructose-1,6-P_2_. In glycolysis, *PK* (ctg1085.g09371) was found upregulated. Within the organic acid pathway, *ACO* (ctg884.g07708), *IDH* (ctg1216.g10454), and *MDH* (ctg1437.g12743) were downregulated, whereas *NADP-ME* (ctg1118.g09496) showed increased expression, together contributing to higher organic acid accumulation in the pulp.

Stage III: The following genes were found upregulated in the sugar metabolism pathway: *INV* (ctg1064.g09177), *SUS* (ctg3154.g27085), *SPS* (ctg468.g03914), and *HK* (ctg183.g01544), collectively promoting rapid sugar accumulation in LR fruits. In glycolysis, *PFK9* (ctg1401.g12237) was upregulated, while in the organic acid pathway, both *MDH* (ctg688.g05954) and *NADP-ME* (ctg1923.g17066) exhibited increased expression.

Stage IV (mature fruit stage): Only the sugar metabolism pathway remained transcriptionally active, with increased expression of *INV* (ctg1064.g09177) and *HK* (ctg183.g01544), suggesting a continued enhancement of hexose phosphorylation during ripening.

Stage V (fully mature stage): In the sugar metabolism pathway, *INV* (ctg938.g08130) was downregulated, while ctg1064.g09177, *HK* (ctg183.g01544), and *FK* (ctg1093.g09429) were upregulated, indicating intensified sucrose cleavage and hexose interconversion in the final maturation phase. In glycolysis, *pckA* (ctg1986.g17876) and *PK* (ctg1085.g09371) were upregulated, whereas *PK* (ctg2432.g21394) was downregulated. Within the organic acid metabolism, *IDH* (ctg1216.g10454) showed reduced expression, suggesting a shift toward decreased organic acid synthesis in fully ripened fruits.

Collectively, these transcriptional patterns highlight a coordinated reprogramming of carbohydrate and organic acid metabolism throughout fruit development. The interplay between *INV*, *SPS*, *SUS*, and *HK* appears central to sugar accumulation, while modulation of *MDH*, *NADP-ME*, and *IDH* expression contributes to the acid balance that shapes the characteristic flavor profile of *B. ramiflora*.

### 3.5 qRT-PCR gene expression analysis

**Figure 6:**
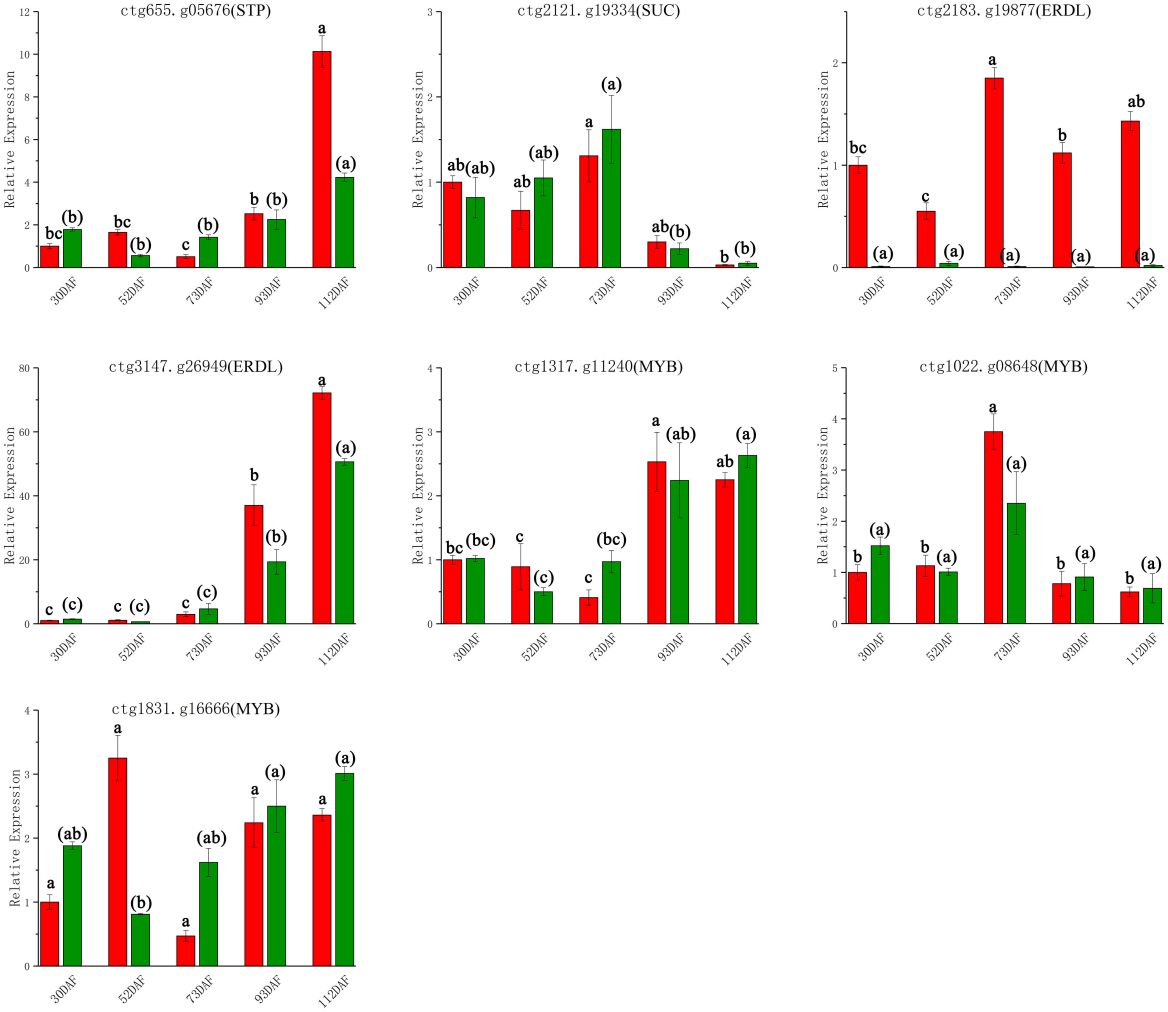
qRT-PCR confirmatory analysis of 7 DEGs Red represents *Baccaurea ramiflora* ‘BR’ and green represents ‘LR’. Different lowercase letters (a, b, c) above the bars indicate statistically significant differences (P < 0.05) as determined by one-way ANOVA followed by Tukey’s HSD test. Bars sharing the same letter are not significantly different, whereas bars with different letters are significantly different.

To validate the transcriptome sequencing results of *B. ramiflora* pulp, 7 DEGs related to the sugar and acid metabolic pathways were selected for qRT-PCR analysis. These genes included *STP12* (ctg655.g05676), *SUC2* (ctg2121.g19334), *ERDL4* (ctg2183.g19877), *ERDL7* (ctg3147.g26949), *MYB61* (ctg1317.g11240), *MYB44* (ctg1022.g08648), and *MYB4* (ctg1831.g16666), with their RNA-seq expression levels detailed in **Supplementary Table S5**. qRT-PCR trends matched RNA-seq (**Figure 6**). This consistency underscores the reliability of the RNA-seq data, ensuring the accuracy of subsequent analyses and enrichment studies on the transcriptomic DEGs in the later stages.

## 4 Discussion

Fruit flavor arises from soluble sugars and organic acids, with other metabolites modulating sweetness, sourness and astringency (Chen et al., 2009; Minas et al., 2013). In recent years, volatile compounds such as esters and alcohols have also been identified as key contributors to flavor formation (Zhang et al., 2020). We found an unusual sugar profile: L−sorbose dominates the pulp, a rare primary sugar in ripe fruits. L−sorbose rose sharply between 70 and 90 DAF, marking ripening onset. Unlike most fruits that accumulate sucrose, glucose and fructose, *B. ramiflora* accumulates L−sorbose. The biochemical significance and potential origin of L-sorbose accumulation. The prevalence of L-sorbose, coupled with the near absence of fructose, points to a unique sugar metabolic network in *B. ramiflora*. L-Sorbose is not a typical intermediate of the central sucrose cleavage pathway. In plants, it is often discussed in the context of L-ascorbic acid (AsA) biosynthesis and metabolism (Wang et al., 2024; Wu et al., 2024). The “Smirnoff-Wheeler” pathway, the major AsA biosynthetic route in plants, proceeds from D-glucose to L-galactose and eventually to L-ascorbic acid. Interestingly, L-sorbose can be formed as an intermediate in alternative AsA biosynthetic pathways or through the modification of existing sugars (Quiñones et al., 2024). Its accumulation may reflect diversion or bottlenecks in AsA−related fluxes or activity of specific epimerases/reductases. Although less sweet than sucrose, high L−sorbose likely contributes to BR sweetness. More importantly, its accumulation, alongside glucose and the absence of fructose, defines a non-canonical sweetness signature for this fruit. This composition may also affect osmotic balance, oxidative stress tolerance and post−harvest traits (Hao et al., 2023). Thus, *B. ramiflora* is a useful model for divergent sugar metabolism and its ecological–physiological implications.

In terms of organic acids, citric acid content was found to be higher during the early to mid-stages of fruit development, followed by a sharp decline at 90 DAF, coinciding with the increasing sugar content. It is important to note that this study specifically focused on the core pathways of sugar and organic acid metabolism. Therefore, the metabolomic analysis was optimized for the identification and quantification of these primary metabolites. Although secondary metabolites such as phenolic acids or flavonoids can also influence fruit flavor, they were not systematically detected or discussed in the current non-targeted metabolomics framework. Furthermore, the lack of analysis of volatile compounds, which are crucial for aroma, and the absence of sensory evaluation data limit our ability to fully interpret how the molecular findings translate into the actual sensory experience. Future research could further explore the contribution of these compounds to the overall flavor profile of *B. ramiflora*. The decline in citric acid at later stages is consistent with the increased sweetness observed in the fruit. Interestingly, while significant differences in glucose concentrations were observed between the LR and BR varieties at full maturity, the citric acid levels showed no significant differences. However, it is important to note that these inferences, drawn from metabolic and transcriptomic data, require direct validation through sensory evaluation and analysis of volatile compounds to confirm that they indeed translate into perceptible differences in taste and aroma. It is noteworthy that in the untargeted metabolomic analysis of this study, free fructose was not detected, which is uncommon in fruits. This could be attributed to the following reasons: First, under the UPLC-MS/MS analysis conditions used in this study, the ionization efficiency of fructose may be lower compared to other sugars that were detected, such as glucose and sorbitol, leading to its signal strength being below the detection limit. More importantly, biological reasons may also contribute. Transcriptomic data indicate that several fructokinase (*FK*) genes are continuously highly expressed during fruit development (**Figure 5**; **Supplementary Table S4**), suggesting that fructose generated from sucrose hydrolysis or other pathways is rapidly phosphorylated and enters downstream sugar metabolism pathways, thereby maintaining an extremely low level of free fructose. This phenomenon suggests that the sweetness composition of B. ramiflora fruit may differ from typical fruits that accumulate sucrose, glucose, and fructose. Instead, it follows a unique sugar accumulation pattern dominated by glucose and sorbitol, which represents its distinctive metabolic characteristics.

Moreover, the sugar-acid metabolic pathways in *B. ramiflora* are intricately linked through the glycolytic pathway, which connects sucrose breakdown to the tricarboxylic acid (TCA) cycle of organic acids (Kubo et al., 2002). In this study, 37 DEGs associated with sugar-acid metabolism pathways were identified, including *INV*, *SUS*, *SPS*, *HK*, *FK*, *GPI*, *FBP*, *PFK9*, *ALDO*, *PGK*, *ENO*, *pckA*, *PK*, *PD*, *ACO*, *IDH*, *MDH*, *NADP-ME*, among others. These genes demonstrate the complexity of the sugar-acid metabolic network and its regulation during fruit development. While these correlations suggest that these genes may play significant roles in sugar metabolism in *B. ramiflora*, it is important to note that correlation does not imply causality. Therefore, further experimental validation is needed to establish their direct roles in sugar accumulation, aligns with observations in some other fruits (Wu et al., 2022).

While the overall pattern of repressed *INV* and induced *SUS*/*SPS* in the sweeter BR variety aligns with observations in some other fruits, our data reveal several species-specific nuances that underlie the unique sugar profile of *B. ramiflora*. Firstly, the differential regulation within gene families is noteworthy. For instance, specific INV isoforms (e.g., ctg1064.g09177) exhibited distinct expression trajectories between BR and LR, suggesting that key paralogs, rather than the entire gene family, drive the phenotypic variation. More importantly, the metabolic context in *B. ramiflora* is distinctive. The near absence of free fructose in the pulp, coupled with sustained high expression of Fructokinase (*FK*) genes, indicates a metabolic channeling where fructose is rapidly phosphorylated rather than accumulated. Therefore, the downregulation of specific INV isoforms in BR not only conserves sucrose but also works in concert with *FK* to shape a unique sugar composition dominated by glucose and L-sorbose, setting it apart from common fruits like apples or citrus. Furthermore, the differential expression of the vacuolar glucose exporter *ERDL7* highlights an additional layer of regulation, potentially facilitating the remobilization of sugars from the vacuole to the cytosol, thereby influencing the cytosolic sugar signaling pool and the overall sugar-acid balance. Thus, the flavor formation in B. ramiflora is not merely a recapitulation of a common theme but is fine-tuned by a combination of isoform-specific enzyme regulation, a distinctive metabolic flux towards non-fructose sugars, and the involvement of specific sugar transporters. Further research into the volatile compounds that contribute to aroma could provide additional insights into the flavor dynamics of this fruit.

To quantitatively address the dynamics of organic acid metabolism, we closely integrated the expression profiles of key TCA cycle genes with the measured citric acid content (**Figure 4**). The sharp decline in citric acid after 70 DAF was strongly correlated with the transcriptional regulation of several critical enzymes. Most notably, the citrate synthase (*CS*, ctg425.g03656) gene, which catalyzes the committed step of citrate synthesis, was significantly downregulated in both varieties at the transition from stage III to IV (70–90 DAF) (**Figure 5**; **Supplementary Table S4**), directly linking reduced synthesis capacity to the observed metabolite depletion. Concurrently, the aconitase (*ACO*, ctg884.g07708) gene, responsible for citrate conversion within the TCA cycle, exhibited an opposing upregulation during this period. This coordinated pattern—repression of *CS* and induction of *ACO*—suggests a metabolic shift away from citrate accumulation and towards its catabolism, a regulatory logic that aligns with findings in other fruits where citrate is remobilized during ripening (Diakou et al., 2000; Kubo et al., 2002). Furthermore, the absence of detectable malic acid, despite the expression of malate dehydrogenase (*MDH*) genes, can be plausibly explained by the consistently high expression of NADP-malic enzyme (*NADP-ME*, e.g., ctg100.g00903 and ctg1207.g10216) (**Figure 5**). *NADP-ME* decarboxylates malate to pyruvate, and its sustained activity likely creates a metabolic “pull” that prevents malate from accumulating to detectable levels, effectively shunting carbon away from the TCA cycle (Hirai and Ueno, 1997). This gene-centric, quantitative analysis moves beyond general pathway description and pinpoints specific transcriptional control points that govern the unique organic acid profile of B. ramiflora pulp. It is also important to consider that the absence of detectable malic acid could be influenced by the sensitivity limitations of the analytical instruments used. The detection threshold of our instruments might not be sufficient to identify malic acid at such low concentrations, which could explain why it was not observed in the analysis. These findings provide further insights into the organic acid metabolism of *B. ramiflora* and highlight the potential for manipulating these pathways to enhance flavor.

Further molecular mechanism studies indicate that there are significant differences in flavor formation between the BR and LR varieties, which may be related to their sugar-to-acid ratio. The higher sucrose accumulation in the BR variety is closely associated with the elevated expression of *SUS* and *SPS* genes, which promotes the formation of sweetness. In contrast, the lower sucrose levels and higher glucose consumption in the LR variety are closely linked to the elevated expression of the *HK* gene, which accelerates glucose metabolism and results in lower sweetness.

This study has a few limitations that should be acknowledged. Due to the relatively small sample size in the current study, the results may not fully represent the genetic and metabolic diversity across different environmental conditions, which could impact the generalizability of the findings. Additionally, the research primarily focused on the analysis of soluble sugars and organic acids, which, while important for understanding fruit flavor, do not encompass all potential contributors to flavor complexity. Crucially, the absence of data on volatile compounds and the lack of formal sensory evaluation represents a significant limitation. For example, volatile compounds, which play a significant role in aroma and overall sensory experience, were not considered in this study. Consequently, while our study identifies pronounced molecular differences between varieties (e.g., in sugar-to-acid ratio and related gene expression), we cannot definitively confirm the extent to which these differences directly explain or predict the actual perceived taste and flavor complexity. The overall flavor perception is a synthesis of taste (sweetness, sourness etc.) and aroma, and the lack of volatile and sensory data means our interpretation of the ‘flavor’ based solely on sugar/acid metrics remains incomplete.

This limitation may prevent a full understanding of the flavor profile of *B. ramiflora*. Future research could benefit from using a larger, more diverse sample size to better capture the genetic and metabolic variability of *B. ramiflora* across different regions and climates. Additionally, incorporating environmental factors such as soil type, temperature, and humidity could provide deeper insights into how these variables influence the metabolic pathways involved in flavor development. Moreover, future studies should consider expanding the scope to include the analysis of volatile compounds and their interactions with sugars and organic acids, as they are crucial for a comprehensive understanding of fruit flavor. The integration of advanced techniques like metabolomics, transcriptomics, and volatile compound profiling could provide a more complete picture of the molecular mechanisms behind flavor development in *B. ramiflora*. This would help identify new targets for breeding strategies aimed at enhancing fruit flavor. By addressing these limitations, future studies will offer a more robust and detailed understanding of the flavor development in *B. ramiflora* and provide valuable information for breeding and cultivation practices to improve the sensory qualities and marketability of this underutilized fruit species.

**Figure 7:**
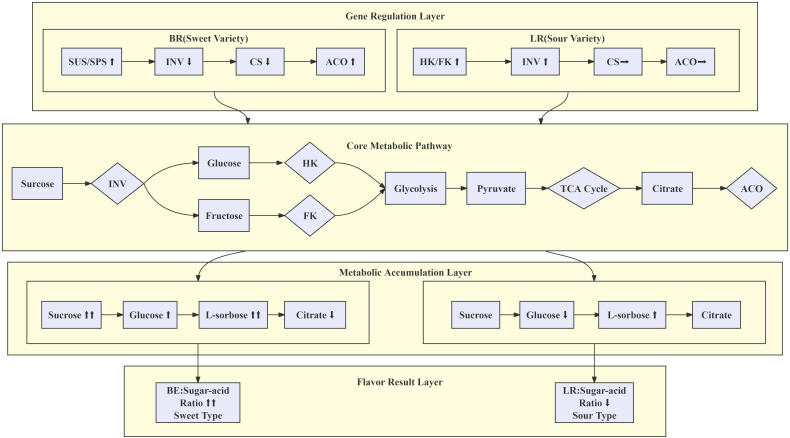
Schematic diagram of sugar and acid metabolism, gene regulation, and flavor outcomes.

The ultimate flavor outcome is an emergent property of the system, determined by the integrated ratio of sugars to acids. In the BR variety, the genetic program of sucrose conservation (low *INV*, high *SUS/SPS*) co-occurs with the metabolic shift to degrade organic acids (low *CS*, high *ACO*). This dual action results in a high sugar-to-acid ratio, manifesting as a sweet taste. In the LR variety, the genetic program of sucrose mobilization and hexose utilization (high *INV* and *HK*) coincides with a less pronounced acid degradation, resulting in a low sugar-to-acid ratio and a sour taste. In summary, our model delineates a clear causal chain: Genetic Regulation (Differential expression of *INV*, *SUS/SPS*, *HK*, *CS*, *ACO*) - Metabolic Phenotype (Accumulation of Sucrose/Glucose/L-Sorbose vs. Citrate; High vs. Low Sugar-to-Acid Ratio) - Sensory Outcome (Sweet vs. Sour Flavor) (**Figure 7**). This framework not only elucidates the molecular basis of flavor variation in *B. ramiflora* but also provides a valuable roadmap for targeted breeding strategies. By selecting for alleles that favor the “sweet” gene expression profile, breeders can effectively steer the metabolic network toward the desired high-sweetness phenotype. The combined analysis of metabolomics and transcriptomics reveals the dynamic regulation of sugar and organic acid levels during fruit ripening, contributing to the distinct sweetness and sourness of the fruit. These findings suggest opportunities for targeted breeding strategies aimed at optimizing flavor profiles through manipulation of key genes such as *INV*, *SUS*, and *SPS*. This approach could lead to the development of new cultivars with improved sweetness and acid balance, thereby enhancing both the sensory quality and marketability of this underutilized fruit species.

## Data Availability

The datasets presented in this study can be found in online repositories. The names of the repository/repositories and accession number(s) can be found in the article/**Supplementary Material**.
